# Regulation of interferon pathway in 2-methoxyestradiol-treated osteosarcoma cells

**DOI:** 10.1186/1471-2407-12-93

**Published:** 2012-03-19

**Authors:** Fritz Wimbauer, Caihong Yang, Kristen L Shogren, Minzhi Zhang, Ribu Goyal, Scott M Riester, Michael J Yaszemski, Avudaiappan Maran

**Affiliations:** 1 Department of Orthopedics, College of Medicine, Mayo Clinic, 3-69 Medical Sciences, Rochester, MN 55906, USA; 2 Paracelsus Medical University, Salzburg 5020, Austria; 3 Tongji Hospital, Tongji Medical College, Huazhong University of Science and Technology, Wuhan 430030, China

**Keywords:** 2-Methoxyestradiol, osteosarcoma, Interferon, ISRE, GAS

## Abstract

**Background:**

Osteosarcoma is a bone tumor that often affects children and young adults. Although a combination of surgery and chemotherapy has improved the survival rate in the past decades, local recurrence and metastases still develop in 40% of patients. A definite therapy is yet to be determined for osteosarcoma. Anti- tumor compound and a metabolite of estrogen, 2-methoxyestradiol (2-ME) induces cell death in osteosarcoma cells. In this report, we have investigated whether interferon (IFN) pathway is involved in 2-ME-induced anti-tumor effects in osteosarcoma cells.

**Methods:**

2-ME effects on IFN mRNA levels were determined by Real time PCR analysis. Transient transfections followed by reporter assays were used for investigating 2-ME effects on IFN-pathway. Western blot analyses were used to measure protein and phosphorylation levels of IFN-regulated eukaryotic initiation factor-2 alpha (eIF-2α).

**Results:**

2-ME regulates IFN and IFN-mediated effects in osteosarcoma cells. 2 -ME induces IFN gene activity and expression in osteosarcoma cells. 2-ME treatment induced IFN-stimulated response element (ISRE) sequence-dependent transcription and gamma-activated sequence (GAS)-dependent transcription in several osteosarcoma cells. Whereas, 2-ME did not affect IFN gene and IFN pathways in normal primary human osteoblasts (HOB). 2-ME treatment increased the phosphorylation of eIF-2α in osteosarcoma cells. Furthermore, analysis of osteosarcoma tissues shows that the levels of phosphorylated form of eIF-2α are decreased in tumor compared to normal controls.

**Conclusions:**

2-ME treatment triggers the induction and activity of IFN and IFN pathway genes in 2-ME-sensitive osteosarcoma tumor cells but not in 2-ME-resistant normal osteoblasts. In addition, IFN-signaling is inhibited in osteosarcoma patients. Thus, IFN pathways play a role in osteosarcoma and in 2-ME-mediated anti-proliferative effects, and therefore targeted induction of IFN signaling could lead to effective treatment strategies in the control of osteosarcoma.

## Background

Osteosarcoma is the most common malignant primary bone tumor in children and adolescents. It is the 6^th ^leading cancer in children under 15. Osteosarcoma accounts for 2.4% of all malignancies in pediatric patients and approximately 20% of all bone cancers [1,2]. Current standard treatment is to use neoadjuvant chemotherapy followed by surgical resection. Although a combination of surgery and chemotherapy has led to improved survival rate, a definite therapy is yet to be determined for this disease. 2-Methoxyestradiol (2-ME), which acts as a potential anti-tumor agent in several types of cancer, has been shown to block cell growth and induce apoptosis in osteosarcoma cells [3-8].

IFNs are a family of cytokines synthesized and secreted by most cell types that elicit pleiotropic biological effects [9]. Three main classes of IFNs namely, α, β and γ have been identified and classified as type I (α, β) and type II (γ). IFNs which induce anti-viral, anti-angiogenic, anti-proliferative, anti-cancer effects and immune modulatory effects, have been shown to block the progression of cancer in vitro and in vivo [9-11]. The biological effects of IFNs are mediated through IFN-regulated genes. Three major IFN-regulated pathways involving RNA-dependent protein kinase (PKR)/the eukaryotic initiation factor (eIF)-2α system, 2-5A synthetase/RNase L system and Janus Kinase (JAK)/signal transducer and activator of transcription (STAT) system have been identified [9]. PKR/eIF2α system mainly mediates type I IFN effects and the protein synthesis factor eIF-2α functions as the effector of PKR-induced effects in this pathway [9]. IFN's role in maintenance therapy has been investigated in osteosarcoma and other cancers. Several studies have demonstrated that IFNs block osteosarcoma growth in patients, animal models, and in cultured cells [10,12]. IFNα has been studied as a maintenance therapy after post-operative chemotherapy with cisplatin, doxorubicin and methotrexate [12,13]. In murine model of osteosarcoma, IFNα reduces the tumor growth and the metastatic nodules in the lungs [14]. IFNβ has been shown to block the growth of osteosarcoma cells in vitro. In addition IFN enhances antitumor activity of chemotherapeutic agents [15,16]. We have demonstrated that the anti-growth effects of 2-ME are accompanied by increases in IFN mRNA levels in osteosarcoma cells [5]. In this report, we have investigated the regulation and association of IFN pathways in 2-ME-mediated effects in osteosarcoma cells.

## Methods

### Primary human osteoblast (HOB) cells

HOB cells were established from cancellous bone obtained as surgical waste material from orthopedic surgical procedures in accordance with a Mayo Clinic Institutional review board (IRB) approved protocol and cultured as explants to generate the osteoblast-like monolayers, as described [17].

### Cell culture

Human osteosarcoma cells (MG63, 143B, KHOS and HOS) and HOB cells were grown in DMEM/F12 medium containing 10% charcoal-stripped fetal bovine serum (FBS), penicillin, streptomycin and geneticin (300 μg/mL) and maintained at 37 C under 5% CO2.

2-ME was purchased from Sigma Chemical Co. (St. Louis, MO). Stock solutions of the metabolites at their respective concentrations were made in 95% ethanol. Human IFN α, β and γ were obtained from Mayo Clinic Pharmacy and suspended in DMEM/F12 medium without serum.

### Reporter assays

The reporter constructs containing γ-activated sequence (GAS) elements, kindly provided by Dr. Aseem Kumar (Laurentian University, Sudbury, Ontario, Canada), interferon-stimulated response element (ISRE) obtained from Stratagene (La Jolla, CA) and IFN-β gene promoter sequences, kindly provided by Dr. Barbara Sherry (North Carolina State University, Raleigh, NC) were used. Osteosarcoma cells plated in 6-well plates (1.5 × 10^6 ^cells/well) were transfected with the indicated reporter luciferase constructs at 60% cell confluence using the transfection reagent Lipofectamine, as described in the manufacturer's protocol (Invitrogen, Carlsbad, CA). IFN-lucifease construct was transfected using the transfection reagent FuGene, as described in the manufacturer's protocol (Promega, Madison, WI). All transfections performed had a control plasmid containing Renilla Luciferase (Promega, Madison, WI) which allowed normalization of luciferase units. Twenty-four hours post transfection, the cells were treated with 2-ME (10 μM) and IFN (2000 units/mL). The cells were harvested after 48 h of treatment or as indicated and suspended in 300 μL of passive lysis buffer provided in a luciferase assay kit (Promega) and read on a TD-20E luminometer (Turner, Sunnyvale, CA).

### RNA isolation and analysis by RT PCR

Cells were plated at 10^6 ^cells per flask in T-75 culture flasks one day prior to metabolite treatment. The next day, cells were replaced with fresh medium containing 10 μM concentrations of 2-ME and incubated for different periods of time. The cells were harvested and the cell pellets were used for RNA isolation. Total cellular RNA was extracted and isolated using a modified organic solvent method, and the RNA yields were determined spectrophotometrically at 260 nm. The levels of IFNα, IFNβ and control actin mRNA were analyzed by Real-time quantitative PCR using ABI 7900HT System (Perkin-Elmer Applied Biosystems, Foster City, CA) as described. The primers used were: IFNα forward: 5'GCTC ACCC ATTT CAAC CAGT3'; IFNα reverse:5' GATG GTTT CAGC CTTT TGGA3'; IFNβ forward:5' GTGT CAGA AGCT CCTG TGGC3'; IFNβ reverse: 5'CTTC AGTT TCGG AGGT AACC3'; Actin forward: 5' TGCCTCAGGGCA3' and Actin reverse: 5'GCTGTGCTATCCCTGTAC3'.

### Normal and osteosarcoma tissues

Osteosarcoma tissues and normal muscle tissues were obtained through a protocol submitted and approved by the institutional review board (IRB), an internal review committee at Mayo Clinic represented by several clinical faculty members including pathologists, oncologists, orthopedic surgeons, legal and administrative team members. The tumor tissues were obtained through surgical resection. The normal muscle tissues collected in this study were normal muscle tissues adjacent to osteosarcoma tissues occurring within the same patient. The histology was reviewed by fellowship-trained musculoskeletal pathologists to confirm proper diagnosis. After collection, the tissues were stored at -70°C until processed for protein analysis.

### Protein analysis and western blot hybridization

Protein analysis was carried out as described [4]. Briefly, vehicle- and 2-ME-treated cells were harvested and lysed by suspending in cell lysis buffer. After centrifugation at 10,000 × g for 10 min, the supernatant was collected, and the protein concentration was determined by Bradford protein assay. Normal and tumor tissues obtained from patients according to the IRB-approved protocol were homogenized in lysis buffer and the protein concentration was determined. Cytoplasmic extract containing protein (60 μg) was analyzed by Western blot hybridization using anti-phosphoeIF-2α (1: 1000 dilution), anti- eIF-2α (1: 3000 dilution) and anti-glyceroldehyde 3-phosphate dehydrogenease (GAPDH) (1: 5000 dilution) antibodies (Santa Cruz Biotechnology, Santa Cruz, CA). The expression levels of proteins on the western blots were quantitated using densitometer and PD Quest 7.4.0 software (BioRad, Hercules, CA).

### Statistical analysis

All values are expressed as means ± standard error. The data is representative of three independent experiments. Significant differences between groups were determined by Fisher's protected least significant difference post hoc test for multiple-group comparisons following detection of significance by one-way analysis of variance (ANOVA). *P *< 0.005 was considered statistically significant.

## Results

### 2-ME effect on IFN gene in osteosarcoma cells

**Figure 1:**
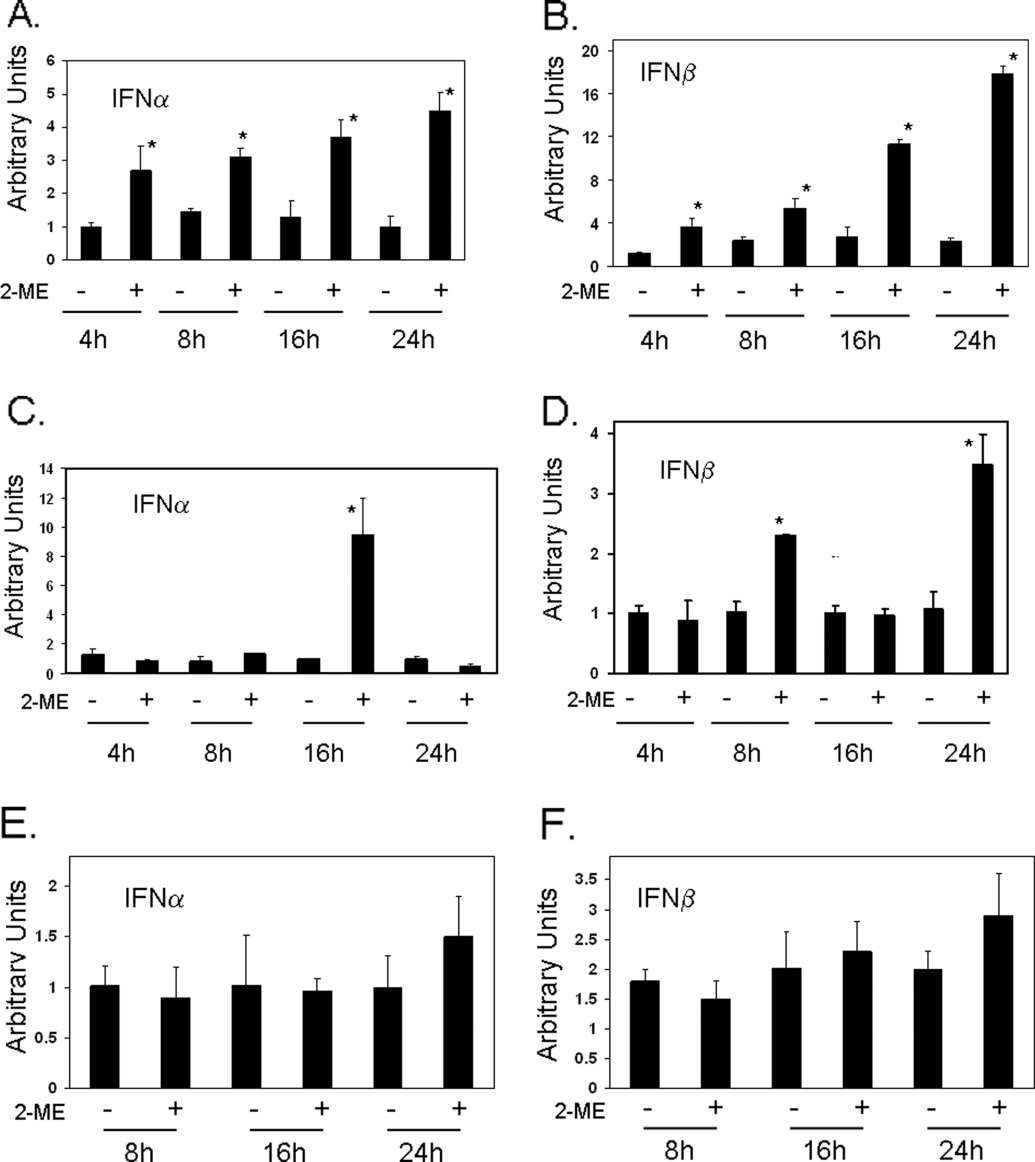
**2-ME effects on IFN mRNA levels**. A)MG63 IFNα mRNA; B) MG63 IFNβ mRNA; C)143B IFNα mRNA; D)143B IFNβ mRNA; E) HOB IFNα mRNA; F) HOB IFNβ mRNA. Cells were treated with Veh and 2-ME (10 μM) for 24 hrs. Total RNA was isolated from cells and analyzed by real time PCR. Values are the mean ± SE(n = 6 replicate cultures) * *P *< 0.05 vs Veh.

We have demonstrated previously that 2-ME treatment induces IFN mRNA levels in MG63 osteosarcoma cells. In order to determine whether 2-ME treatment affects IFN mRNA levels in osteosarcoma cell types with different metastatic potentials, we have carried out Real time PCR and measured on IFN mRNA levels in MG63 and 143B osteosarcoma cells. 2-ME treatment induced IFNα and β mRNA levels in MG63 osteosarcoma cells (Figure 1A and 1B). IFNα mRNA levels were increased by 2.7-, 2-, 3- and 4.5-fold, and IFNβ mRNA levels were increased by 3-, 2-, 4- and 7.8-fold at 4, 8, 16 and 24 h, respectively. 2-ME increased IFNα mRNA levels at 16 h by 8-fold compared to vehicle control but did not have any effect at other time points (8 and 24 h) analyzed (Figure 1C). 2-ME treatment induced IFNα and β mRNA levels in 143B osteosarcoma cells (Figure 1C and 1D). 2-ME increased IFNα mRNA levels at 16 h by 8- fold compared to vehicle control but did not have any effect at other time points (8 and 24 h) analyzed (Figure 1C). 2-ME treatment increased IFNβ mRNA levels at 8 and 24 h by 2- and 3.5-fold and did not affect at 16 h compared to the vehicle controls (Figure 1D). Our results show that 2-ME does not regulate IFNα and β mRNA levels in normal osteoblasts (Figure 1E and 1-F).

**Figure 2:**
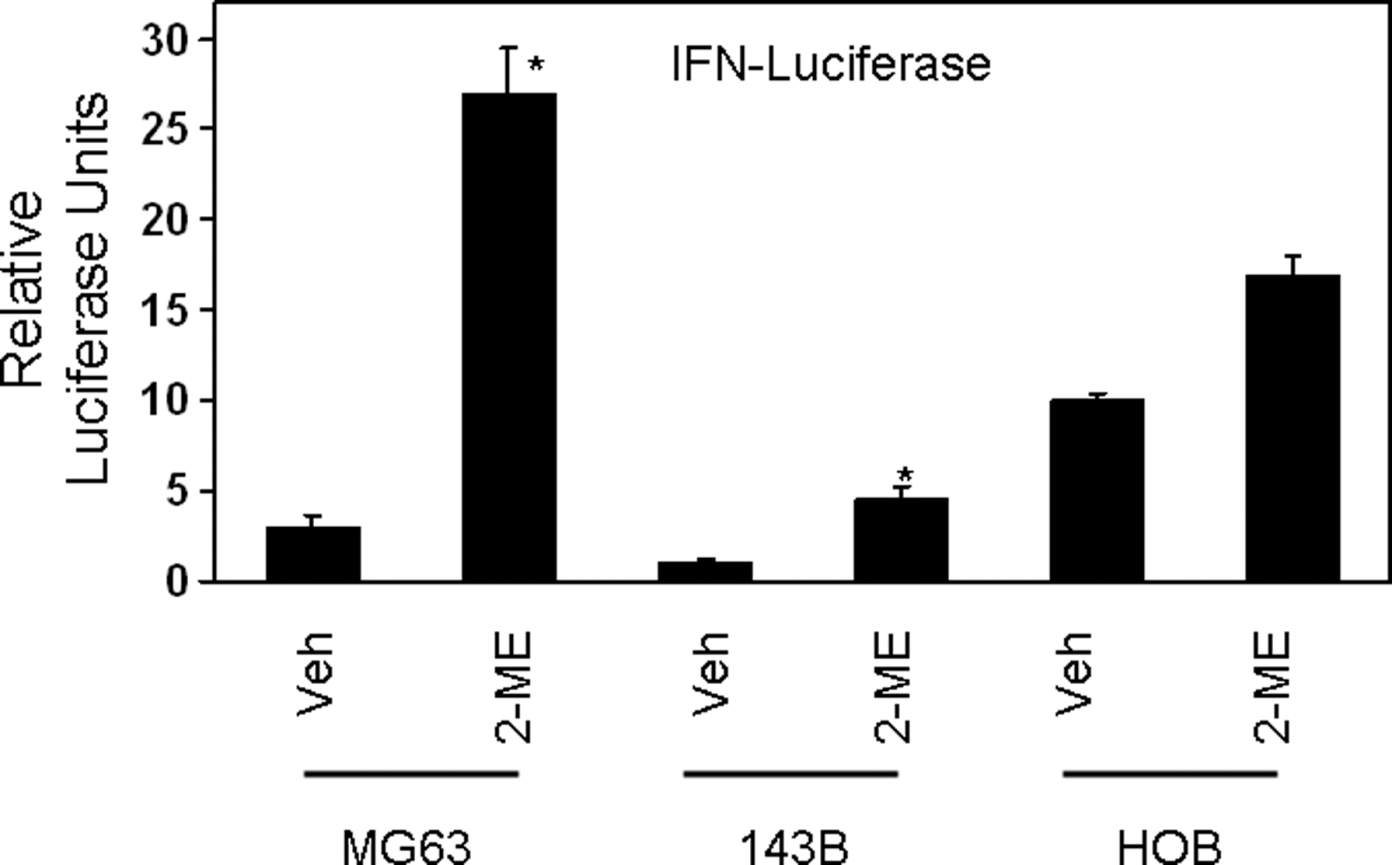
**2-ME effect on IFN gene promoter Activity in Osteosarcoma Cells**. MG63 and 143B cells transiently transfected with pblueIFNluciferase were treated with Veh and 2-ME (10 μM) for 24 hrs and the luciferase reported activity was analyzed. Values are the mean+ SE(n = 6 replicate cultures).

In order to determine whether 2-ME has a direct effect on IFN genes, we have investigated the effect of 2-ME on IFNβ gene-driven luciferase activity by transient transfection assays in human osteosarcoma cells. Relative to vehicle control, 2-ME treatment stimulated the luciferase activity by 9- and 4.5-fold in MG63 and143B osteosarcoma cells, respectively (Figure 2). Our results show that 2-ME does not regulate IFNβ gene-driven luciferase activity in normal HOB cells (Figure 2).

### 2-ME effects on IFN-dependent effects in osteosarcoma cells

**Figure 3:**
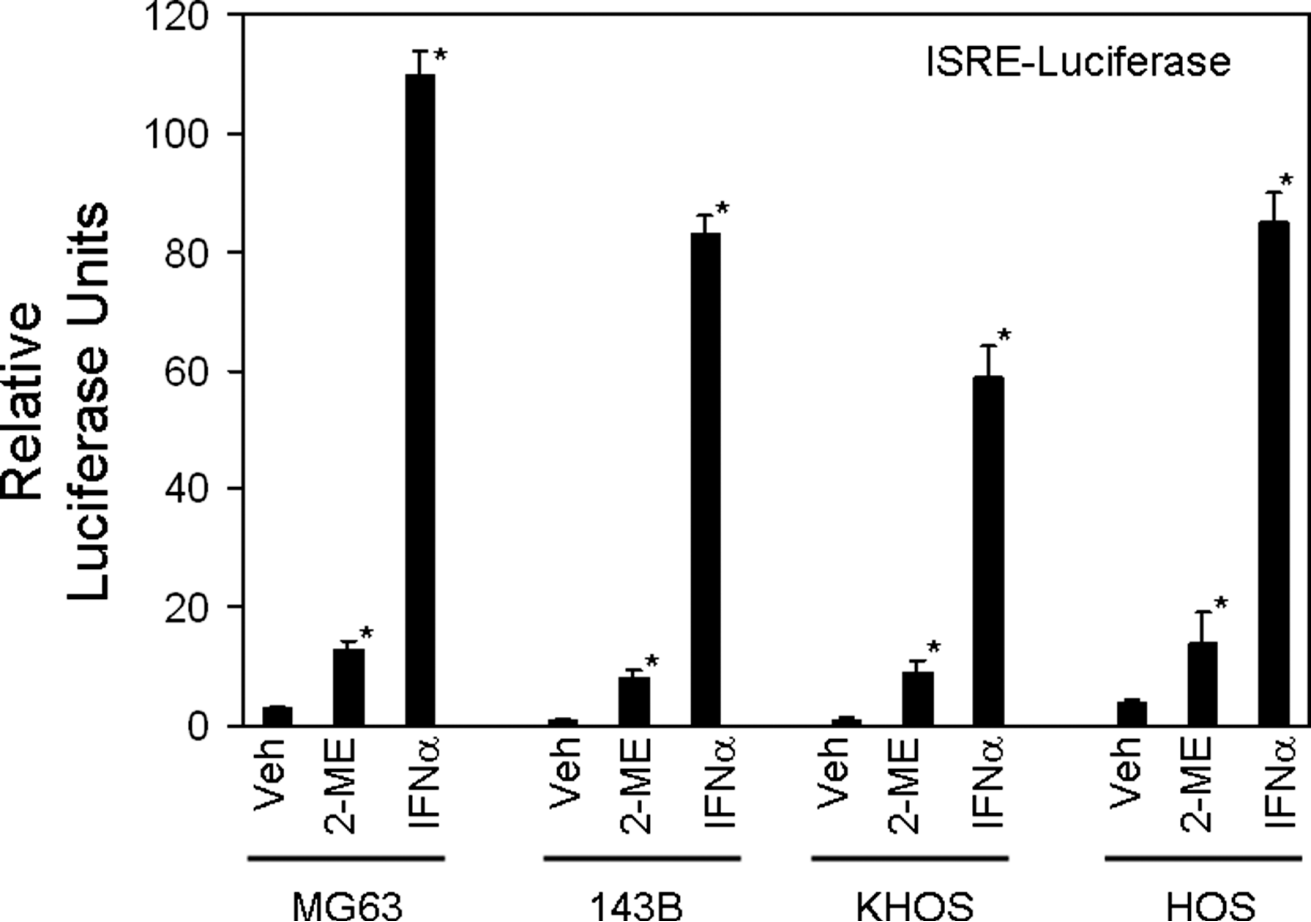
**2-ME effect on ISRE-Luciferase Activities in Osteosarcoma Cells**. MG63 and 143B cells transiently transfected with ISRE-luciferase were treated with Veh and 2-ME (10 μM) for 24 hrs and the luciferase reporter activity was analyzed. Values are the mean+ SE(n = 6 replicate cultures) * *P *< 0.05 vs Veh.

**Figure 4:**
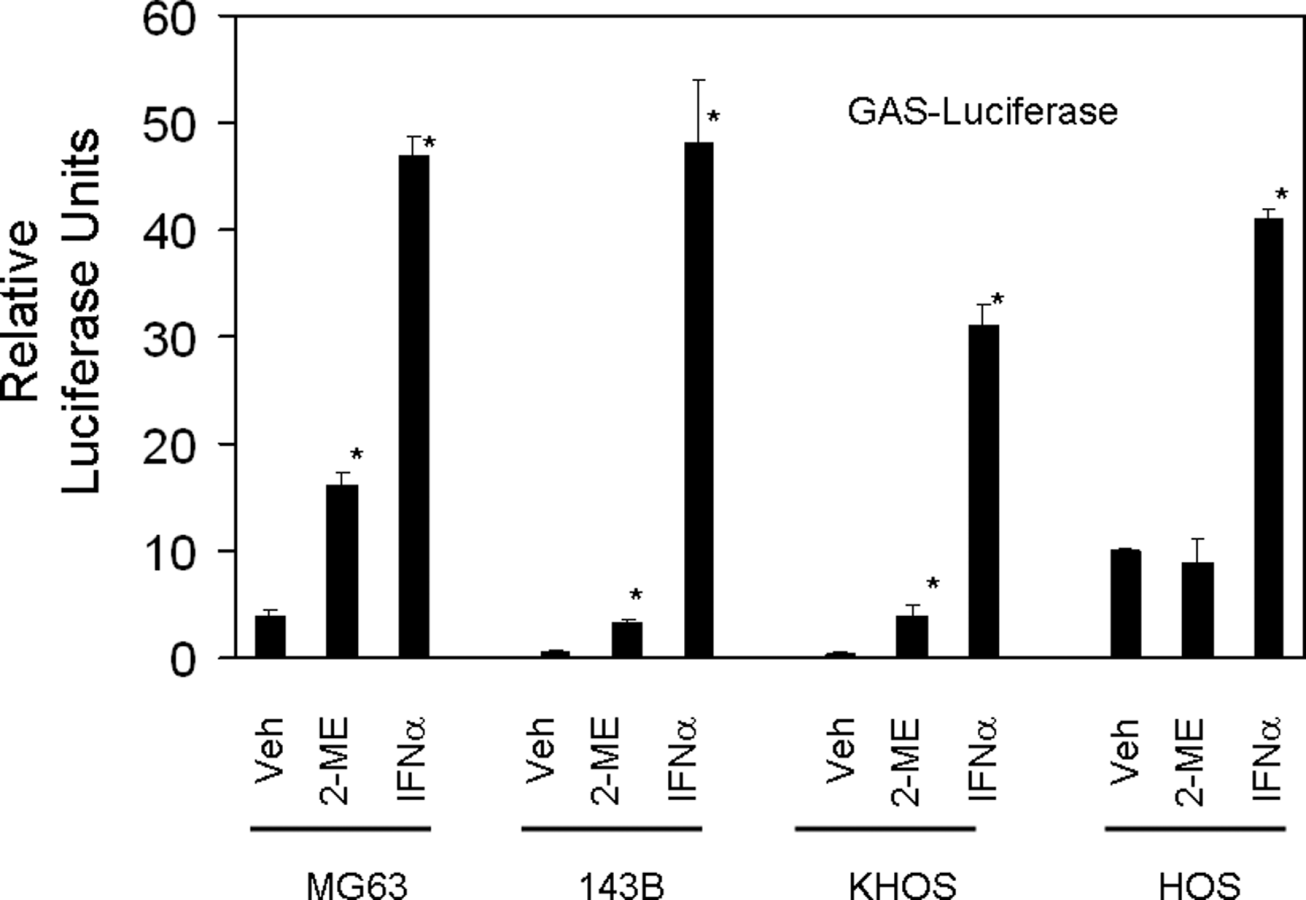
**2-ME effect on GAS-Luciferase Activities in Osteosarcoma Cells**. Cells transiently transfected with GAS-luciferase were treated with Veh and 2-ME (10 μM) for 24 hrs and the luciferase reporter activity was analyzed. Values are the mean+ SE(n = 6 replicate cultures) * *P *< 0.05 vs Veh.

**Figure 5:**
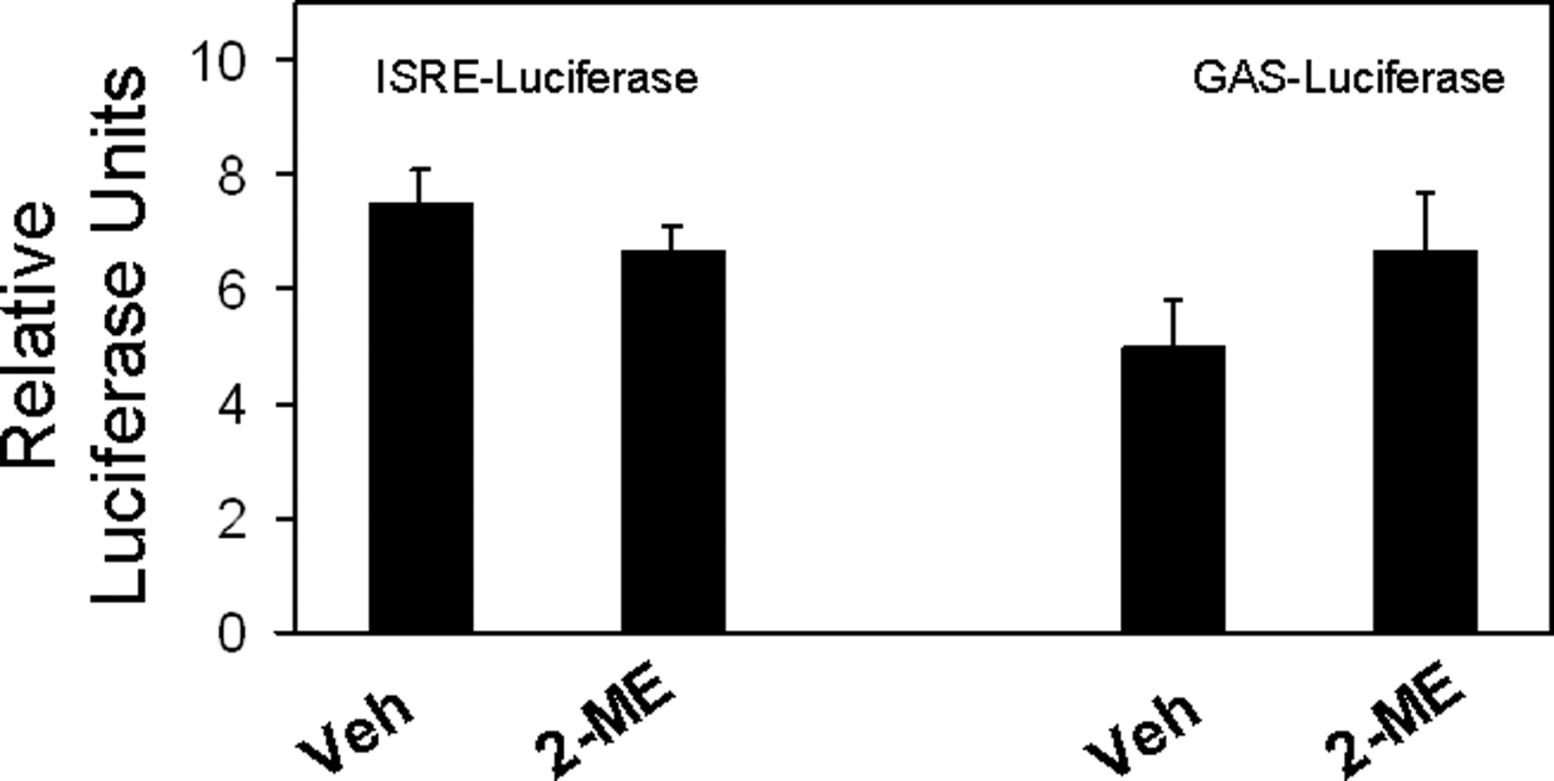
**2-ME effect on GAS- and ISRE-activities in normal osteoblasts**. HOB cells transiently transfected with GAS- and ISRE-luciferase were treated with Veh and 2-ME(10 μM) for 24 hrs and the luciferase reporter activity was analyzed. Values are the mean+ SE(n = 6 replicate cultures).

To determine whether 2-ME treatment induces IFN-dependent transcription, we have tested the effect of 2-ME treatment on interferon-stimulated response element (ISRE) and γ-activated sequence (GAS). 2-ME actions on luciferase reporter constructs driven by ISRE and GAS sequences were determined by transient transfection assays in human osteosarcoma cells. 2-ME treatment induced IFN-stimulated response element (ISRE) sequence-dependent transcription by 4-, 8-, 9- and 4.5-fold in MG63, 143B, KHOS and HOS cells, respectively (Figure 3). 2-ME stimulated gamma-activated (GAS) sequence-dependent transcriptions by 4-, 5- and 10-fold in MG63, 143B and KHOS cells, respectively (Figure 4). 2-ME, however, showed no effect on GAS-dependent transcription in HOS osteosarcoma cells. Our studies from normal osteoblasts shown in Figure 5 indicate that both ISRE and GAS-dependent luciferase activities are not altered in primary HOB cells after transient expression of ISRE-and GAS-luciferase plasmid constructs followed by treatment of 2-ME indicating that 2-ME treatment does not affect ISRE- and GAS-dependent transcriptions in normal osteoblasts.

### 2-ME effect on IFN-regulated eukaryotic initiation factor (eIF)-2α

**Figure 6:**
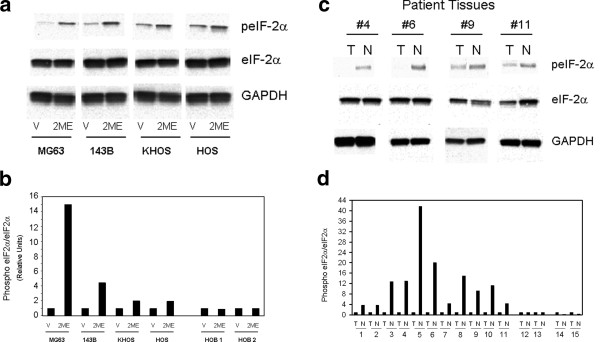
**Analysis of eIF-2α and phospho eIF-2α protein levels**. Western blot from osteosarcoma cells (**A**) and 4 representative osteosarcoma tissues (**C**); Quantitation of densitometry signals from osteosarcoma cells (**B**) and osteosarcoma patient tissues (**D**). The cytoplasmic extracts prepared from Vehicle control (Veh) and (10 μM) 2-ME-treated cells, and from normal (N) and osteosarcoma tumor (T) tissues were analyzed by western blot hybridization using anti-eIF2α, anti-phophoeIF2α and anti-GADPH antibodies. The bands were analyzed by quantitative densitometry. After western blot analysis, the signals from the corresponding bands were quantitated and the ratio of peIF-2α over eIF-2α was determined after normalization to GAPDH.

To determine the effect of 2-ME on eIF-2α, the effector of IFN-regulated anti -proliferative and anti-tumor signals we have investigated the protein extracts from vehicle and 2-ME-treated osteosarcoma cells. Our results indicate that the eIF-2α phosphorylation increases in the presence of 2-ME treatment. 2-ME treatment increases eIF-2α phosphorylation after 16 h by 15-, 4, 2- and 2-fold in MG63, 143B, KHOS and HOS cells compared to vehicle (Figure 6A &6B) [18]. 2-ME treatment does not affect eIF-2α and phosphor eIF-2α in HOB cells (Figure 6A). Our results show that the levels of non-phosphorylated eIF-2α and the control glyceraldehyde 3-phosphate dehydrogenase (GAPDH) are not affected in the presence of vehicle control and 2-ME-treatment (Figure 6A).

### Regulation of eIF-2α protein in osteosarcoma patients

Several experimental studies and clinical trials show that osteosarcoma responds to IFN therapy. To investigate whether IFN pathway is regulated in osteosarcoma, we have analyzed the regulation of eIF-2α protein. Figure 6 shows the levels of phosphorylated and total levels of eIF-2α from four representative normal control muscle and osteosarcoma tissues from 15 patients. The representative blot from four tissues is shown in Figure 6C. Quantitation of signals from protein analysis by western blot hybridization shows that ratio of peIF-2α to eIF- 2α decreases from 3- to 42-fold in 11 of 15 osteosarcoma tissues analyzed, demonstrating an increased phosphorylation of eIF-2α in normal compared to tumor (Figure 6D). The phosphorylation of eIF-2α is increased by 3-fold and 9-fold in 2 tumor tissues compared to normal and does not alter in 2 patients analyzed. Whereas, the levels of non-phosphorylated eIF-2α and the control glyceraldehyde 3-phosphate dehydrogenase (GAPDH) are not affected in control and tumor tissues (Figure 6C).

## Discussion

Unlike the parent compound, 17β-estradiol, the metabolite 2-ME shows anti-growth effects and functions as a potential therapeutic agent in several types of cancer. We have previously shown that 2-ME induces cell death in osteosarcoma cells but not in normal osteoblasts [4,5]. In this report, we demonstrate that 2-ME regulates IFN pathway in a number of osteosarcoma cell lines. 2-ME induces IFN gene promoter activity, expression, and IFN-dependent transcriptional activities in low tumorigenic MG63 and high tumorigenic 143B osteosarcoma cells. 2-ME dependent effects on IFN genes and pathways are specific to osteosarcoma cells and are associated with anti-proliferative effects. Whereas 2-ME does not influence IFN and IFN-dependent pathways in normal HOB cells which have been previously shown to be resistant to the anti-growth activities of 2-ME [5]. Our results also show that the eIF-2α protein and the downstream effectors of IFN pathway are regulated by 2-ME treatment in several osteosarcoma cell lines. In addition, eIF-2α phosphorylation is decreased in osteosarcoma tumor patients compared to normal. Our results suggest that IFN pathway is involved in osteosarcoma growth and regulation.

It has been reported earlier that 2-ME-mediated cell death is accompanied by increases in IFNβ mRNA levels in low metastatic MG63 osteosarcoma cells [5]. Current investigation shows that 2-ME-induces type I IFN (α and β) mRNA levels in low and high tumorigenic osteosarcoma cells. In MG63 cells, the mRNA levels increase at 16 and 24 h. Whereas, in 2-ME-treated 143B cells, IFNα increases at 16 h and IFNβ mRNA increases at 8 and 24 h. While this biphasic induction needs to be studied further, the late induction may be due to secondary responses and to transcription factors that may be activated during early response. The induction of mRNA appears to be due to the induction of gene promoter activity. 2-ME-mediated induction of IFN promoter suggests that 2-ME might recruit transcription factors (proteins) that could directly bind to regulatory elements on the IFN promoter. Previous studies show that (nuclear factor kappa B) NFκB, (interferon regulatory factor) IRF-1 and P53 proteins directly bind to IFN gene sequences and induce transcription. 2- ME-mediated anti-tumor effects in osteosarcoma cells and in several other systems involve the induction of P53 [6,19]. Further work is needed to determine whether P53 or other protein transcription factors are involved in these biological effects of 2-ME and contribute to 2-ME-stimulated responses and IFN promoter activities.

IFN-mediated transcriptional activation and signaling has been well studied by several investigators using various model systems [9]. IFN actions trigger a cascade of events leading to ISRE and GAS dependent transcriptional events [20,21]. While ISRE effect is mainly induced through the type I IFNs, IFNα and IFNβ [22], the GAS effects are induced by type II IFN family member, IFNγ. [23]. Our results show that 2-ME treatment increased transcription of ISRE- dependent transcription in MG63, 143B, KHOS and HOS osteosarcoma cells. Similarly, GAS-dependent transcription is increased in MG63, 143 and KHOS osteosarcoma cells but not in HOS cells. This differential response could be due in part to the differences in cell origin and the genetic heterogeneity of osteosarcoma. GAS and ISRE activations are due to specific activation of JAK/STAT family members and formation of dimeric complexes of STAT family members in cytoplasm and translocation into nucleus [9,20,24]. However, detailed investigations are required to identify the nature of hetero- and homo-dimers that might be forming in 2-ME-treated cells and contributing to the activation of ISRE and GAS elements.

2-ME treatment does not affect IFN promoter activity or gene expression and IFN-regulated pathways in normal HOB cells. We have previously demonstrated that 2-ME induces anti-growth activities in several osteosarcoma cell lines but not in normal primary HOB cells [5]. Current studies suggest that there is an association between 2-ME-induced anti-proliferative effects and the induction of IFN pathways in 2-ME-treated osteosarcoma cells.

IFN was approved nearly three decades ago for clinical applications. IFN-α2 remains a mainstay of treatment for viral infections and certain forms of cancer including osteosarcoma. Studies have shown that the protein products of IFN, the couple of hundred IFN stimulated genes (ISGs), underlie the biological and clinical effects of IFNs [9,11]. They provide fundamental cellular defense mechanisms against viral infections and cancer. Studies from other groups have shown that co-treatment of IFN improves the anti-tumor effects of chemotherapeutic agents such as doxorubicin and etoposide [15]. The European American oncology study group (EURAMOS) is investigating the effect of IFN as a follow up therapy in patients who have undergone conventional chemotherapy[10,12]. Furthermore, IFN has been shown to increase the chemotherapy sensitivity of osteosarcoma cell lines [25]. Although additional work is warranted to delineate these molecular pathways in 2-ME treated osteosarcoma cells, our findings point out that IFN signaling could play important role in the control of osteosarcoma.

IFN-regulated anti-tumor mechanisms are mediated by eukaryotic initiation factor(eIF)-2, and through the down-regulation of protein synthesis [26,27]. EIF-2-mediated mechanism has been well documented in IFN-treated cells and in several other cancer models [27]. EIF-2 forms a ternary complex with GTP and MET-tRNA and delivers the initiation factor tRNA to the ribosomal site of protein synthesis [26,28-30]. The eIF-2 is discharged and released as a complex with GDP. This GDP should be replaced with GTP in order for eIF-2 to form a new ternary complex and trigger a new set of translation initiation. The phosphorylated form of eIF-2 is not able to trigger initiation resulting in the inhibition of protein synthesis [26-28]. Our work from previously published reports and from current studies indicate that 2-ME treatment results in the phosphorylation of eIF-2α protein in vitro. The regulation is specific to anti-growth activities of 2-ME since 2-ME does not appear to regulate eIF-2α in HOB cells. Our analyses of tissue specimens from patients show decreased levels of the phosphorylated form of eIF-2α protein in osteosarcoma tissues compared to normal (Table 1). The disease specific regulation was observed in 11 out of 15 specimens. The eIF-2α phosphorylation is associated with osteosarcoma independent of organ and histology subtype as summarized in Table 1. This raises the possibility that a cause for tumor progression could be the decreased eIF-2α phosphorylation which eventually results in decreased inhibition of protein synthesis and apoptosis in tumor cells. Additional data involving a large number of tissues would be necessary to conclude whether the apparent difference in phosphorylation reflects the apparent differences in growth regulation between normal and tumor tissues. These observations further corroborate our findings that exposure to 2-ME leads to increased phosphorylation of eIF-2α resulting in increased apoptotic and anti-tumor effects due to the inhibition of protein synthesis in osteosarcoma cells [18]. These investigations point out that decreased IFN-signaling could be potentially associated with the disease state in osteosarcoma tissues. Recent studies show that IFN inhibitory activity in osteosarcoma patients could be an important factor that contributes to tumor progression. Inhibition of IFN pathways could lead to increased susceptibility to disease and progression of disease to metastatic stages [31]. These findings and our current results emphasize that understanding of IFN signaling could help diagnose and provide optimal therapy in osteosarcoma patients.

**Table 1:** Analysis of phospo and non-phospo eIF-2α protein levels in osteosarcoma tissues

Tumor Tissue sample	PhosphoeIF-2α/.eIF-2α ratio (fold change*)	diagnosis	Age at resection	Tumor site
1	-1	Fibroblastic osteosarcoma	20	Femur
2	-4	Chondroblastic osteosarcoma	18	Pelvis
3	-13	Chondroblastic osteosarcoma	13	Pelvis
4	-13	Chondroblastic osteosarcoma	39	Sacrum
5	-42	Osteogenic osteosarcoma	21	Sacrum
6	-20	Chondroblastic osteosarcoma	40	Sacrum
7	-4	Chondroblastic osteosarcoma	54	Scapula
8	-15	Osteogenic oseosarcoma	20	Femur
9	-9	Osteogenic osteosarcoma	11	Femur
10	-11	Chondroblastic osteosarcoma	13	Scapula
11	-4	Osteosarcoma, lung metastasis	57	Pelvis
12	0	Osteogenic osteosarcoma	15	Tibia
13	0	Fibroblastic osteosarcoma	17	Femur
14	+10	Fibroblastic osteosarocma	53	Pelvis
15	+3	Osteogenic osteosarcoma	54	Femur

* The values represent the changes compared to corresponding normal samples.

## Conclusions

In conclusion, we have shown that IFN signaling is associated with osteosarcoma regulation and 2-ME-mediated cell death in osteosarcoma cells. Hence, upregulation of IFN pathway could be an effective strategy for controlling osteosarcoma.

## Competing interests

Mayo Clinic has a user patent on 2-methoxyestradiol in applications involving bone cancer.

## Authors' contributions

FW carried out the transfections and reporter assays. CY and RG carried out the cell treatment and western blot analysis. MZ performed the RNA analysis. KS carried out the reporter assays, western blot analysis, coordinated the study and performed the statistical analysis. SR coordinated the patient tissue collection and data analysis. MJY contributed to the study design and clinical data analysis. AM designed the experiments, coordinated the study, interpreted the results, and drafted the manuscript. All authors read and approved the final manuscript.

## Pre-publication history

The pre-publication history for this paper can be accessed here:

http://www.biomedcentral.com/1471-2407/12/93/prepub
